# Angiotensin converting enzyme gene polymorphism is associated with severity of coronary artery disease in men with high total cholesterol levels

**DOI:** 10.1007/s13353-012-0083-3

**Published:** 2012-02-04

**Authors:** Joanna Borzyszkowska, Anna Stanislawska-Sachadyn, Marcin Wirtwein, Wojciech Sobiczewski, Dariusz Ciecwierz, Radoslaw Targonski, Marcin Gruchala, Andrzej Rynkiewicz, Janusz Limon

**Affiliations:** 1 Department of Biology and Genetics, Medical University of Gdansk, Debinki 1, 80-211 Gdansk, Poland; 2 First Department of Cardiology, Medical University of Gdansk, Debinki 7, 80-211 Gdansk, Poland; 3 Department of Invasive Cardiology, Medical University of Gdansk, Debinki 7, 80-211 Gdansk, Poland

**Keywords:** *ACE*, *AGTR1*, Atherosclerosis, CAD, *CYP11B2*, Gensini score

## Abstract

This study examines whether renin-angiotensin-aldosterone system gene polymorphisms: *ACE* (encoding for angiotensin converting enzyme) c.2306-117_404 I/D, *AGTR1* (encoding for angiotensin II type-1 receptor) c.1080*86A>C and *CYP11B2* (encoding for aldosterone synthase) c.-344C>T are associated with the extension of coronary atherosclerosis in a group of 647 patients who underwent elective coronary angiography. The extension of CAD was evaluated using the Gensini score. The polymorphisms were determined by PCR and RFLP assays. The associations between genotypes and the extent of coronary atherosclerosis were tested by the Kruskal-Wallis test, followed by pairwise comparisons using Wilcoxon test. The population has been divided into groups defined by: sex, smoking habit, past myocardial infarction, BMI (>, ≤ 25), age (>, ≤ 55), diabetes mellitus, level of total cholesterol (>, ≤ 200 mg/dl), LDL cholesterol (>, ≤ 130 mg/dl), HDL cholesterol (>, ≤ 40 mg/dl), triglycerides (>, ≤ 150 mg/dl). Significant associations between the *ACE* c.2306-117_404 I/D polymorphism and the Gensini score in men with high total cholesterol levels (P_Kruskal-Wallis_ = 0.008; P_adjusted_ = 0.009), high level of LDL cholesterol (P_Kruskal-Wallis_ = 0.016; P_adjusted_ = 0.028) and low level of HDL cholesterol (P_Kruskal-Wallis_ = 0.04; P_adjusted_ = 0.055) have been found. No association between the *AGTR1* c.1080*86A>C and *CYP11B2* c.-344C>T and the Gensini score has been found. These results suggest that men who carry *ACE* c.2306-117_404 DD genotype and have high total cholesterol, high LDL cholesterol and low HDL cholesterol levels may be predisposed to the development of more severe CAD.

## Introduction

Coronary artery disease (CAD) is a major public health problem in many industrialized countries and remains the leading cause of death in most of them. The renin-angiotensin-aldosterone system (RAAS) is a well-described hormone system that regulates blood pressure and salt/water homeostasis. RAAS dysfunction contributes to functional and structural perturbations that occur in atherosclerosis formation (Farmer and Torre-Amione 2001).

The studies investigating the relation between the RAAS gene polymorphisms and CAD progression are designed either to search for an association between the polymorphisms and the atherosclerosis severity represented by the chosen scale of arterial narrowing (e.g. Gensini, jeopardy, Leaman’s, number of vessels narrowed at least in 50% or 75%), which is the approach we applied, or to evaluate the CAD risk associated with the presence of specific genotype in case-control studies, the approach that prevails.

The most studied and clinically important polymorphism in the angiotensin converting enzyme gene (*ACE*) is an insertion/deletion (I/D) of a 288 bp *Alu* repeat sequence within the intron 16 (*ACE* c.2306-117_404, rs4340). *ACE* c.2306-117_404 I/D polymorphism has been suggested to be related to approximately 50% of the variability in the ACE levels in the bloodstream, with the highest values present among DD homozygotes (Rigat et al. 1990). Further, the *ACE* c.2306-117_404 I/D polymorphism has been widely investigated as a CAD risk factor (Zintzaras et al. 2008; Agerholm-Larsen et al. 2000). An association between this polymorphism and the extent of coronary atherosclerosis has been reported for populations ranging from 152 to 1162 participants (Ye et al. 2003; Mendonca et al. 2004; Niemiec et al. 2008a; Hibi et al. 1997), although other studies did not report such an association (Jeunemaitre et al. 1997; Nakauchi et al. 1996; Qiu et al. 2007; Foy et al. 1997; van Geel et al. 2001).

The 1166A>C substitution within the 3‘ untranslated region of the angiotensin II type-1 receptor gene (*AGTR1* c.1080*86A>C, rs5186) occurs in a *cis*-regulatory region recognized by a specific microRNA-155 (miR-155) (Martin et al. 2007). For the C allele, the base-pairing complementarity of miR-155 and the *AGTR1* transcript is interrupted, thus decreasing the ability of miR-155 to interact with the cis-regulatory site, which results in miR-155 being no longer an attenuator of translation, which further leads to a higher expression of AGTR1 (Martin et al. 2007). It has been demonstrated that AGTR1 expression is significantly increased in the CC homozygotes in comparison to AA and AC genotype carriers (Ceolotto et al. 2011). The *AGTR1* c.1080*86A>C polymorphism has been investigated as a CAD risk factor (Xu et al. 2010). Further, the correlation between the *AGTR1* c.1080*86A>C polymorphism and the extension of CAD has been found by some (Nakauchi et al. 1996; Qiu et al. 2007) (study populations of 133 and 130 participants, respectively), but not by all investigators (Gardemann et al. 1998; van Geel et al. 2001; Jeunemaitre et al. 1997) (study populations ranging from 463 to 2244 participants).

The aldosterone synthase gene (*CYP11B2*) polymorphism (*CYP11B2* c.-344C>T, rs1799998) is located within the steroidogenic factor-1 (SF-1) binding site, thus SF-1 binding capability has been suggested to vary between the alleles, although this hypothesis has not been finally confirmed (White and Rainey 2005) and thus the potential biological functionality needs to be yet determined. To our knowledge, the association between the *CYP11B2* c.-344C>T polymorphism and the extent of atherosclerosis in CAD has not been reported; however the *CYP11B2* c.-344C>T polymorphism has been related to the severity of atherosclerotic plaque size in the carotid artery (Sharma and Katz 2010). Previously the relationship between the *CYP11B2* c.-344C>T polymorphism and hypertension (Sookoian et al. 2007) has been extensively studied. Also, to date, either the T (Casiglia et al. 2005) or C allele (Tsukada et al. 2002) have been reported to be risk factors in cardiovascular events. However, a study finding no relation has also been published (Payne et al. 2004).

The aim of the present study was to examine whether three common RAAS genes polymorphisms: *ACE* c.2306-117_404 I/D, *AGTR1* c.1080*86A>C and *CYP11B2* c.-344C>T are associated with the extension of coronary atherosclerosis, represented by the Gensini score, which is a reliable estimate of the severity of coronary artery stenosis (Gensini 1983), in a relatively large cohort of 647 Polish-Caucasian patients undergoing elective coronary angiography.

## Materials and methods

### Subjects

The patients were admitted to the First Department of Cardiology, Medical University of Gdansk, Poland, between 2005 and 2007 for elective coronarography due to CAD. The patients were on pharmacological treatment prior to the coronarography. Further, only those patients who had undergone first-time coronary angiography for suspected CAD were included. The resultant study population consisted of 647 consecutive patients (368 males, 279 females).

Fasting blood samples were tested for levels of: total cholesterol, high-density lipoprotein (HDL) cholesterol and triglycerides (TG) with the use of standard enzymatic-colorimetric methods. The low-density lipoprotein (LDL) cholesterol level was calculated according to the Friedewald formula. Smoking habit (smoker, non-smoker, ex-smoker) was self-reported by study participants. All subjects were Polish Caucasians and gave their written informed consent to take part in this study. The study was approved by the Ethical Committee of the Medical University of Gdansk, Poland.

### Estimation of the severity of coronary stenosis

The patients underwent coronary angiography in accordance with standard protocols. The severity of CAD was evaluated by Gensini scores determined by examining coronary angiograms. Gensini score is a point scale, which is based on the number of stenotic coronary artery segments including degree of its luminal narrowing and the localization of the stenosis (Gensini 1983). Thus the Gensini score is calculated as a sum of stenosis scores and functional significance scores calculated for each segment of the coronary artery tree. Stenosis score expresses the percentage of the reduction in the diameter of the coronary artery lumen (the score was given from 1 to 32 for complete occlusion). The functional significance score illustrates the regional importance of the lesion’s position (the score was given from 0.5 to 5). Gensini score equals 0 if there is no stenotic change in any of the coronary arteries segment whereas the maximal value is achieved for theoretical total occlusion in the main segments of the coronary artery tree.

### Genetic analyses

Genomic DNA was isolated from peripheral blood lymphocytes in accordance with a standard phenol-chloroform method.

A polymerase chain reaction (PCR) assay was performed to detect the *ACE* c.2306-117_404 I/D polymorphism. The primer sequences were modified from Rigat et al. (1992): forward (5‘-CTGGAGACCACTCCCATCCTT-3’), reverse (5‘-GATGTGGCCATCACATTCGTC-3’). PCR reactions were performed in a final volume of 10 μl. Each PCR tube contained: 30 ng of genomic DNA template, 0.2 μM of each primer, 200 μM of each dNTP, 2 μM MgCl_2_, 1 μl of 10 × PCR buffer (Fermentas, Lithuania) and 0.5 U of *Taq* DNA polymerase (Fermentas, Lithuania). The thermocycling conditions were as follows: 95°C for 5 min, 30 cycles of 95°C for 30 s, 63°C for 1 min and 72°C for 2 min, followed by final extension at 72°C for 15 min. The shorter D allele could be preferentially amplified and thus, to avoid mistyping of ID heterozygotes as DD homozygotes, whenever a DD genotype was found a confirmatory PCR reaction was performed, with primers specific for the insertion sequence: (forward 5‘-TGGGACCACAGCGCCCGCCACTAC-3’) and reverse (5‘-TCGCCAGCCCTCCCATGCCCATAA-3’) (Lindpaintner et al. 1995). The second PCR was performed with the same conditions except the inclusion of 10% dimethylsulfoxide (DMSO). The first PCR products were: 490 bp (for I allele) and 202 bp (for D allele). The second PCR products were: 337 bp (for I allele). PCR products were separated in 2% agarose gels, stained with ethidium bromide.

The *AGTR1* c.1080*86A>C polymorphism was detected by PCR/restriction fragment length polymorphism (RFLP) assay. The PCR primer sequences were as follows: forward (5‘-GGATGTATTGATTCAACTAGGCATC-3’), reverse (5‘-AAAGTCGGTTCAGTCCACATAATGC-3’). PCR reactions were performed in a final volume of 20 μl and contained 60 ng of genomic DNA template, 0.2 μM of each primer, 200 μM of each dNTP, 2 μM MgCl_2_, 2 μl of 10 × PCR buffer (Fermentas, Lithuania) and 0.75 U of *Taq* DNA polymerase (Fermentas, Lithuania). The thermocycling conditions were as follows: 95°C for 5 min, 35 cycles of 95°C for 30 s, 58°C for 30 s and 72°C for 1 min, followed by 72°C for 10 min. The 437 bp PCR products were digested with 4 U of *Hpy*F3I (*DdeI* isomer) restriction endonuclease (Fermentas, Lithuania) for 18 h at 37°C. The resulting restriction products were: 240 bp, 200 bp for the AA genotype; 240 bp, 200 bp, 140 bp, 60 bp for the AC genotype and 240 bp, 140 bp, 60 bp for the CC genotype. PCR and RFLP products were separated in 3% agarose gels, stained with ethidium bromide.

The *CYP11B2* c.-344C>T polymorphism was detected by PCR/RFLP assay. The PCR primer sequences were as follows: forward (5‘-CTGTGGTGGAGGGTGTACCT-3’), reverse (5‘-TCCAGGGCTGAGAGGAGTAA-3’). PCR reactions were performed in a final volume of 25 μl. Each PCR tube contained: 30 ng of genomic DNA template, 0.4 μM of each primer, 200 μM of each dNTP, 1.5 μM of MgCl_2_, 2.5 μl of 10 × PCR buffer (Fermentas, Lithuania) and 0.5 U of *Taq* DNA polymerase (Fermentas, Lithuania). The thermocycling conditions were as follows: 95°C for 5 min, 35 cycles of 95°C for 30 s, 59°C for 30 s and 72°C for 20 s, followed by 72°C for 7 min. PCR products were digested with 2 U of *Bsu*RI (*Hae*III isomer) restriction enzyme (Fermentas, Lithuania) with the inclusion of bovine serum albumin (BSA) for 18 h at 37°C. The resulting restriction products were: 124 bp, 60 bp for the CC genotype; 184 bp, 124 bp, 60 bp for the TC genotype and 184 bp for the TT genotype. PCR and RFLP products were separated in 3% agarose gels, stained with ethidium bromide.

### Statistical analyses

The Hardy-Weinberg equilibriums for each of the genotypes tested were calculated using the chi square test. The relation of the analyzed polymorphisms and the extent of coronary atherosclerosis were compared among the groups by the Kruskal-Wallis test, followed by the Wilcoxon analyses for comparisons of two groups. *P*-values were corrected for multiple comparisons using Bonferroni method, when appropriate. The level of statistical significance was set at *P* < 0.05. All statistical analyses were carried out using the SAS 9.2 package (SAS Institute Inc., Cary, NC, USA).

## Results

Clinical and laboratory data of CAD patients including the atherosclerotic risk factors are summarised in Table 1. The Gensini score equals 0 for 242 patients meaning no significant stenosis in their coronary arteries visualized by coronary angiography was found.

**Table 1:** Clinical and laboratory data of 647 patients with CAD

		Gensini score*		
		All	Males	Females
		10 (0-48)	26 (0-61)	0 (0-28)
		(647)	(368)	(279)
Smoke	non-smoker	0 (0-31)	18 (0-53)	0 (0-16)
		(240)	(75)	(165)
	ex-smoker	20 (0-56)	28 (2-68)	4 (0-40)
		(304)	(220)	(84)
	smoker	18.5 (0-52)	32 (2-62)	5 (0-32)
		(84)	(62)	(22)
Past MI	No	2 (0-30)	9.5 (0-42)	0 (0-14)
		(433)	(222)	(211)
	Yes	40 (11-86)	46 (22-97)	28 (0-56)
		(213)	(145)	(68)
BMI	≤25	10 (0-48)	31 (2-58)	0 (0-27)
		(213)	(113)	(100)
	>25	10 (0-48)	25 (0-66)	0 (0-28)
		(434)	(255)	(179)
Age	≤55	2 (0-32)	18 (0-52)	0 (0-16)
		(233)	(87)	(146)
	>55	20 (0-56)	28 (2-69)	4 (0-40)
		(414)	(281)	(133)
Diabetes mellitus	No	8 (0-47)	25 (0-60)	0 (0-14)
		(495)	(282)	(213)
	Yes	25.5 (3-52.5)	29 (5-62)	20.5 (0-44)
		(152)	(86)	(66)
Total cholesterol	≤200 mg/dl	8 (0-44)	24 (0-58)	0 (0-20)
		(409)	(249)	(160)
	>200 mg/dl	16 (0-52)	32 (7-62)	3 (0-40)
		(238)	(119)	(119)
LDL cholesterol	≤130 mg/dl	9 (0-46)	27 (0-64.5)	0 (0-20)
		(482)	(288)	(194)
	>130 mg/dl	14 (0-49)	22.5 (4-56)	5 (0-41)
		(165)	(80)	(85)
HDL cholesterol	>40 mg/dl	14 (0-53)	30 (0-67.5)	0 (0-32)
		(205)	(124)	(81)
	≤40 mg/dl	9 (0-47)	23 (0-57.5)	0 (0-25)
		(442)	(244)	(198)
TG	≤150 mg/dl	7 (0-44)	25 (0-58)	0 (0-22)
		(473)	(261)	(212)
	>150 mg/dl	20 (0-56)	30 (6-62)	4 (0-41)
		(174)	(107)	(67)

*Median (25th-75th quartile)(N)

In the population as a whole, the distributions of the *ACE* c.2306-117_404 I/D, *AGTR1* c.1080*86A>C and *CYP11B2* c.-344C>T genotypes did not significantly differ from that expected by Hardy-Weinberg equilibrium. The distributions of Gensini scores were skewed and could not be normalized by several transformation calculations. Therefore non-parametric and ranked analyses were chosen.

We found no association between the *ACE* c.2306-117_404 I/D, *AGTR1* c.1080*86A>C and *CYP11B2* c.-344C>T and the severity of coronary artery lesions in CAD patients in the population as a whole.

The potential associations of examined polymorphisms and the severity of the disease were examined within groups created by subdividing the population according to: sex, smoking habit, past myocardial infarction (MI), BMI (>25, ≤25), age (>55, ≤55), diabetes mellitus, total cholesterol (>200 mg/dl, ≤200 mg/dl), LDL cholesterol (>130 mg/dl, ≤130 mg/dl), HDL cholesterol (>40 mg/dl, ≤40 mg/dl), TG (>150 mg/dl, ≤150 mg/dl) levels.

A significant association between the *ACE* c.2306-117_404 I/D polymorphism and the extent of coronary atherosclerosis was found in men with high total cholesterol levels (>200 mg/dl) (P_Kruskal-Wallis_ = 0.008; P_IIvsDallel_ = 0.0023; P_adjusted_ = 0.009), and also in men with high LDL cholesterol levels (>130 mg/dl) (P_Kruskal-Wallis_ = 0.016; P_IIvsDallel_ = 0.007; P_adjusted_ = 0.028) and low HDL cholesterol levels (≤40 mg/dl) (P_Kruskal-Wallis_ = 0.04; P_IIvsDallel_ = 0.0138; P_adjusted_ = 0.055), with the highest values of Gensini score present in the *ACE* c.2306-117_404 D allele carriers in comparison to other genotypes (Table 2). No significant association between the *ACE* c.2306-117_404 I/D polymorphism and the extent of coronary atherosclerosis was found when the population was analyzed in other subgroups, as listed above (data not presented).

**Table 2:** Median Gensini score values defined by the *ACE* c.2306-117_404 I/D genotypes in the whole population and in subsets

	Genotype:	Gensini scale*			*P*_Kruskal-Wallis_
		*ACE*	*ACE*	*ACE*	
		DD	ID	II	
Whole investigated population		10 (147)	10 (305)	8.5 (180)	0.92
Sex	Males	25.5 (84)	31 (173)	23 (100)	0.26
	Females	0 (63)	0 (132)	2.5 (80)	0.49
Total cholesterol ≤200 mg/dl	All	4 (83)	8 (205)	14 (112)	0.24
	Males	9.5 (50)	25 (122)	28.5 (70)	0.29
	Females	0 (33)	0 (83)	2.5 (42)	0.46
Total cholesterol >200 mg/dl	All	26.5 (64)	17 (100)	4.5 (68)	0.09
	Males	37.5 (34)	35 (51)	7 (30)	**0.008** ^a^
	Females	3 (30)	0 (49)	2.5 (38)	0.74
LDL cholesterol ≤130 mg/dl	All	4 (103)	9.5 (236)	10.5 (132)	0.42
	Males	16 (61)	32 (138)	28.5 (80)	0.32
	Females	0 (42)	0 (98)	1 (52)	0.52
LDL cholesterol >130 mg/dl	All	32 (44)	16 (69)	5.5 (48)	0.07
	Males	37 (23)	25 (35)	7 (20)	**0.016** ^b^
	Females	6 (21)	4 (34)	4.5 (28)	0.75
HDL cholesterol >40 mg/dl	All	3 (38)	17.5 (106)	26 (56)	0.32
	Males	26 (21)	30 (67)	38 (33)	0.34
	Females	0 (17)	2 (39)	0 (23)	0.78
HDL cholesterol ≤40 mg/dl	All	11 (109)	9 (199)	6 (124)	0.54
	Males	25 (63)	31.5 (106)	10 (67)	**0.04** ^c^
	Females	0 (46)	0 (93)	3 (57)	0.24

*Median (N)

^a^P_IIvsDD_ = 0.0056 for comparison of *ACE* c.2306-117_404 II vs. DD; P_IDvsDD_ = 0.91 for ID vs. DD; P_IDvsII_ = 0.008 for ID vs. II, P_IIvsDallel_ = 0.0023 for II vs. D allel

^b^P_IIvsDD_ = 0.007 for comparison of *ACE* c.2306-117_404 II vs. DD; P_IDvsDD_ = 0.51 for ID vs. DD; P_IDvsII_ = 0.03 for ID vs. II, P_IIvsDallel_ = 0.007 for II vs. D allel

^c^P_IIvsDD_ = 0.08 for comparison of *ACE* c.2306-117_404 II vs. DD; P_IDvsDD_ = 0.60 for ID vs. DD; P_IDvsII_ = 0.014 for ID vs. II, P_IIvsDallel_ = 0.0138 for II vs. D allel

P_Kruskal-Wallis_ = 0.018 for the association between the *CYP11B2* c.-344C>T polymorphism and the Gensini score was found in women with high LDL cholesterol levels (>130 mg/dl). However, after Bonferroni corrections for multiple comparisons, P-value was no longer statistically significant (P_CCvsTallel_ = 0.045; P_adjusted_ = 0.18) (data not presented). No significant associations were found between the *CYP11B2* c.-344C>T polymorphism and the Gensini score in other subgroups either (data not presented).

No significant associations between the *AGTR1* c.1080*86A>C polymorphism and the severity of coronary artery lesion in CAD patients were found in any of the subgroups analyzed (data not presented).

## Discussion

The prominent role of the RAAS in the maintenance of cardiovascular homeostasis suggests that allelic variants of genes encoding for the components of this hormone system could modulate the severity of CAD. This study evaluates the association of three RAAS gene polymorphisms: *ACE* c.2306-117_404 I/D, *AGTR1* c.1080*86A>C, *CYP11B2* c.-344C>T and the extension of coronary lesions, represented by Gensini score, which is based on the number of stenotic coronary artery segments, and a degree of the luminal narrowing and the regional importance of the lesion’s position in the coronary tree (Gensini, 1983), and thus comprises the real reflection of the severity of the CAD.

The results we present demonstrate an association between the *ACE* c.2306-117_404 I/D polymorphism and the extension of atherosclerosis assessed by the Gensini score, with values ≥0. The association was found among men with high total cholesterol levels (>200 mg/dl), high LDL cholesterol levels (>130 mg/dl) and low HDL cholesterol levels (≤40 mg/dl). Our results suggest that pro-atherosclerotic actions of angiotensin II are more pronounced when enhanced by other systemic mechanisms, such as a long-lasting exposure to common CAD risk factors.

The importance of ACE in CAD etiology is undisputed. The chronic influence of higher levels of angiotensin II in the *ACE* D allele carriers, in comparison to the I allele carriers (Jeunemaitre 2008), may concur to the development and/or progression of atherosclerosis. The enhanced ACE expression in macrophages and smooth muscle cells of coronary artery plaques has been previously reported to indicate that ACE activity in lesions contributes greatly to the progression of atherosclerosis (Ohishi et al. 1997).

Our results correspond to a few previous studies. An association between the *ACE* c.2306-117_404 D allele and the severity of CAD, described by the number of narrowings of 50% or higher and by the number of critical arterial occlusions has been reported in a Polish population (Niemiec et al. 2008a), analyzed as a whole, of 172 young (30-55 years old) subjects diagnosed with CAD. The scale chosen by Niemiec et al. (2008a) to assess the severity of CAD does not include localization of the narrowing involved in the Gensini scale but requires including at least one severe arterial narrowing. In a population of 296 patients, Mendonca et al. (2004) showed that the DD genotype is significantly linked to the severity of CAD, which has been assessed by the Leaman’s score measuring the number of arteries with more than 75% stenosis and thus accounts for severe changes only. In a population of 1162 subjects Ye et al. (2003) found that among patients who carry D allele CAD has been more extensive. The severity of atherosclerosis was defined in their study as the number of coronary segments with 5-75% stenosis so patients with greater atherosclerotic extension were excluded. Also, the results of meta-analysis (Sayed-Tabatabaei et al. 2003) investigating the association between the *ACE* c.2306-117_404 I/D polymorphism and the carotid artery intima-media thickness (IMT), used to detect the presence and to follow the progression of atherosclerosis, correspond with our work. Its authors report a positive association between the D allele and the carotid IMT, with the association being stronger among high-risk patients.

Our finding is both sex-specific and related to metabolic factor. Also, among 11 studies, which included sex interaction, described in recent meta-analysis (Zintzaras et al. 2008), six reported positive association between the *ACE* c.2306-117_404 I/D polymorphism and CAD in males only. Zintzaras et al. (2008) suggested the actions of corticosteroid hormones affecting the activity of the RAAS might be involved. Freitas et al. (2008) demonstrated that the presence of DD genotype increases the risk of developing CAD when associated to each of classical risk factors: hypertension, obesity, diabetes and hyperlipidemia considered as total cholesterol ≥200 mg/dl, TG ≥150 mg/dl, LDL cholesterol ≥130 mg/dl and HDL cholesterol ≤40 mg/dl. Investigating a case-control study, Niemiec et al. (2007) concluded that the presence of the D allele increases the risk of CAD associated with the presence of hyperlipidemia defined by total cholesterol ≥5 mmol/l, LDL cholesterol ≥3 mmol/l, TG ≥1,7 mmol/l and overweight/obesity. Hibi et al. (1997) found that the *ACE* c.2306-117_404 I/D polymorphism correlates with the number of vessels with stenosis greater than 75%, with the number of lesions with stenosis greater than 50% and extent index, calculated on the basis of percentage of the segment’s length that appeared abnormal, among smokers. However, the authors did not find an association within the group with hyperlipidemia defined as total cholesterol ≥5.7 mmol/l.

Our findings contrast with those from several studies. It is, however, important to note that the comparisons between the studies may not be accurate as various scales were applied for the measurement of atherosclerosis progression. No associations between the *ACE* c.2306-117_404 polymorphism and the atherosclerosis extent measured by Gensini score, by the number of coronary arteries with stenosis greater than 75% (Nakauchi et al. 1996) and using other scales (Qiu et al. 2007; Foy et al. 1997; Jeunemaitre et al. 1997; van Geel et al. 2001) have been reported.

We did not find any associations between the *AGTR1* c.1080*86A>C polymorphism and the severity of CAD as determined by Gensini score either within the whole population or in any of the investigated subgroups. Such a result is supported by a recent meta-analysis (Xu et al. 2010) which includes 53 studies. The authors conclude that there is only a weak association between the *AGTR1* c.1080*86A>C polymorphism and coronary heart disease. Similarly, no association between the *AGTR1* c.1080*86A>C polymorphism and the Gensini score (Gardemann et al. 1998; Nakauchi et al. 1996), number of arteries stenosed (Gardemann et al. 1998; van Geel et al. 2001) and the angiographic score (Jeunemaitre et al. 1997) has been found. Interestingly, Nakauchi et al. (1996) report that among the patients with the AC genotype the number of arteries narrowed by 75% was higher than among those carrying the AA genotype. Further, Qiu et al. (2007) found that the *AGTR1* c.1080*86A>C polymorphism may be involved in the severity of CAD, with the number of stenosed vessels and the coronary jeopardy score greater in the patients with the AC genotype than in those with the AA genotype.

Our results are in agreement with those reported by Freitas et al. (2008), who did not find any association between the *AGTR1* c.1080*86A>C polymorphism and the increased risk of CAD, also in the context of dislipidemia, defined as total cholesterol ≥200 mg/dl, TG ≥150 mg/dl, LDL cholesterol ≥130 mg/dl and HDL cholesterol ≥40 mg/dl. However, Niemiec et al. (2008b) found that the C allele is associated with a higher risk of CAD in patients with hypercholesterolemia (total cholesterol level ≥5 mmol/l).

The association between the *CYP11B2* c.-344C>T polymorphism and the extent of coronary atherosclerosis has not been investigated before in patients who had undergone first-time coronary angiography. In our study, this polymorphism has not been associated with the extent of coronary atherosclerosis directly or via interactions with commonly known atherosclerotic risk factors. Interestingly, the *CYP11B2* c.-344C>T polymorphism has been associated with the progression of atherosclerotic plaque size in the carotid artery (Sharma and Katz 2010). Our results are in agreement with previous investigations reporting no association between the *CYP11B2* c.-344C>T polymorphism and bypass degradation measured by adjusted Gensini score (Ortlepp et al. 2001), stent restenosis (Ryu et al. 2002), caroid IMT (Balkestein et al. 2002; Sarzani et al. 2003) and overall CAD events risk in 2490 healthy men (Payne et al. 2004). Therefore, further studies regarding the association between the *CYP11B2* c.-344C>T polymorphism and atherosclerosis progression would be of interest.

## Conclusions

In this study, we investigated the association between the *ACE* c.2306-117_404 I/D, *AGTR1* c.1080*86A>C and *CYP11B2* c.-344C>T polymorphisms, within the genes encoding for RAAS proteins, and the extent of atherosclerosis. The association was searched in groups of patients, selected according to common CAD risk factors, such as abnormal lipids profile. Our results suggest that those men who carry the *ACE* c.2306-117_404 DD genotype and who have high total, high LDL or low HDL cholesterol levels may be more predisposed to the development of more severe CAD. No association between the *AGTR1* c.1080*86A>C or *CYP11B2* c.-344C>T polymorphisms and the extent of atherosclerosis in CAD has been found.
